# The Impact of Micronutrient Fortified Foods on Cognitive Functioning among Low-Income Children: A Pilot and Feasibility Study

**DOI:** 10.3390/nu12113351

**Published:** 2020-10-30

**Authors:** Juliana F. W. Cohen, Kelly Sagar, Mary Kathryn Dahlgren, Laura B. F. Kurdziel, Staci A. Gruber

**Affiliations:** 1 Department of Public Health and Nutrition, Merrimack College, 315 Turnpike Street, North Andover, MA 01845, USA; 2 Department of Nutrition, Harvard T.H. Chan School of Public Health, 677 Huntington Ave, Boston, MA 02115, USA; 3 Cognitive and Clinical Neuroimaging Core, McLean Hospital, Belmont, MA 02478, USA; ksagar@mclean.harvard.edu (K.S.); dahlgren@mclean.harvard.edu (M.K.D.); gruber@mclean.harvard.edu (S.A.G.); 4 Department of Psychiatry, Harvard Medical School, Boston, MA 02115, USA; 5 Department of Psychology, Merrimack College, North Andover, MA 01845, USA; kurdziell@merrimack.edu

**Keywords:** cognitive functioning, executive functioning, child and adolescent diet, micronutrients

## Abstract

Brain development continues throughout childhood and requires micronutrients for optimal maturation, but studies have typically examined only a limited number of micronutrients and there has been inconsistent use of validated cognitive measures. This study evaluated the impact of providing low-income children with a daily fortified meal (570 kcal) in the form of a bar and shake containing >75% of the FDA Daily Values for all essential vitamins and minerals, as well as macronutrients (e.g., omega-3 and omega-6 fatty acids and protein), in an afterschool care setting (instead of the usual meal provided) on cognitive functioning. Students aged 8–12 were randomly assigned to intervention (*n* = 19) or control (*n* = 16) meals. Students completed the Stroop Color Word Task, Trail Making Test, and Conner’s Continuous Performance Task (CPT) at baseline and 3 months post-intervention. Differences in cognitive scores were examined using 2 × 2 mixed model ANOVAs (Stroop and CPT) and ANCOVAs (Trail Making Test). Significant main effects of time indicated improvements in both intervention and control groups, but there were no significant main effects of group or group*time interactions. When the amount of meal consumed was examined, most results became non-significant, suggesting that overall meal consumption significantly impacted the observed results. Overall, this pilot study suggests that there may be limited additional benefits to short-term consumption of micronutrient fortified meals among low-income children in an afterschool care setting, and potential benefits observed may be directly related to the amount of food consumed.

## 1. Introduction

Within the United States, the majority of children do not consume diets that align with the Dietary Guidelines for Americans [1]. In general, children consume diets that are too high in sugar, saturated fats, and refined grains and too low in fruits, vegetables, and milk [1,2,3,4]. As a result of these poor dietary patterns, many nutrients are under-consumed, including potassium, dietary fiber, choline, magnesium, calcium, iron, and vitamins A, D, E, and C [5]. Children from low-socioeconomic households are at particular risk of poor dietary quality and insufficient micronutrient intake patterns [6,7], which may have important health implications as they are associated with increased risk of being overweight, type 2 diabetes, and risk factors for cardiovascular disease [4,8,9]. Importantly, evidence is also emerging that poor diets may negatively impact children’s cognitive functioning [10].

Brain development (e.g., synapse formation, neurogenesis, and myelination) continues throughout childhood and late adolescence and requires micro- and macronutrients for optimal maturation [11,12,13]. Supplementation trials examining individual nutrients have primarily focused on iron and iodine, as well as zinc, B vitamins, and omega-3 fatty acids, as playing a particularly important role in supporting children’s brain development [14]. Additionally, synergistic effects of nutrients may impact children’s cognitive functioning as well [15]. However, studies that have examined supplementation with multiple micronutrients on cognitive functioning have had mixed results [15]. This may be due to only select micronutrients being tested, interactions between nutrients, or that supplements alone may be less beneficial than whole foods, which contain more than just vitamins and minerals. Studies examining food quality in children in developed countries have reported preliminary evidence that healthier food consumption (such as fruits, vegetables, fish, and whole grains) is associated with better cognitive functioning, while less healthy food consumption (e.g., red/processed meats, sugary beverages, and snacks with refined grains) is associated with poorer cognitive functioning [10]. However, the majority of studies examining children’s food consumption have not been conducted in controlled environments, and there is generally inconsistent use of validated cognitive measures.

Given these gaps in knowledge, this pilot study was designed to evaluate the impact of providing low-income children with a fortified meal containing all essential vitamins and minerals, omega-3 and omega-6 fatty acids, protein, and fruit on several measures of cognitive functioning, in particular those focused on attention and executive functioning. We hypothesized that compared to children consuming standard less healthy meals typically provided to students, children eating enhanced meals would show improvements in cognitive functioning using validated cognitive tests.

## 2. Materials and Methods

### 2.1. Participants

**Figure 1:**
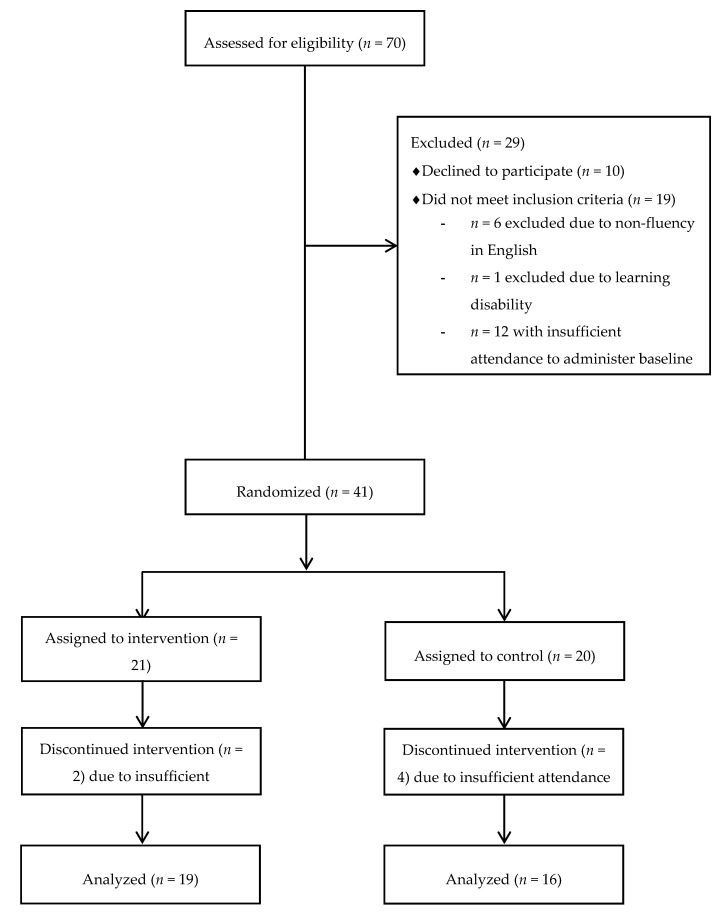
Participant Enrollment Flow Chart.

Students aged 8–12 years participating in an afterschool program at a YMCA in a low-income, urban area in Massachusetts were recruited for this study. This afterschool program was selected because it was located in a region with one of the highest rates of poverty and food insecurity in the state [16] and because it focused on supporting vulnerable (e.g., low-income and food insecure) children in this community. Among the 70 students in the program, 10 declined to participate and 60 returned a parent consent form, student assent form, and a questionnaire completed by parents/guardians. The questionnaire included information on child demographics (i.e., age, sex, race/ethnicity), food allergies, and study eligibility including information on serious medical conditions (e.g., diabetes and/or other chronic medical conditions), learning disabilities (e.g., attention deficit hyperactivity disorder, disabilities that required accommodations for testing, and/or were in special education), English fluency, and attendance at the afterschool program. Out of 60 students, a total of 41 children met eligibility criteria and were randomized to intervention (*n* = 21) or control (*n* = 20) status. Participants were randomly assigned using a simple randomization procedure (computerized random numbers) to 1 of 2 treatment groups (1:1 allocation). However, as six students did not have regular attendance at the afterschool program during the study period, only 35 students (intervention *n* = 19 and control *n* = 16) were included in the final analyses (Figure 1).

### 2.2. Study Meals

Students were randomly assigned to receive either standard (control) meals or intervention meals. The standard meals were those regularly provided by the YMCA afterschool program, which typically consisted of juice and processed meats with refined grains (e.g., hot dog on a bun, sandwich with deli meats, etc.). As there are likely multiple micro- and macro-nutrients that potentially impact cognitive functioning (including potentially synergistic effects), students in the intervention group were given both bars and shakes that were fortified with all essential vitamins and minerals, omega-3 and omega-6 fatty acids, and protein (Nutrient Foods brand [Supplemental Table S1]). As there may be additional benefits from whole foods beyond vitamins and minerals, and to enhance the palatability of the intervention meals, the shakes were also made with fresh/frozen fruits (i.e., blueberries, cherries, bananas, peach, mango, pineapple, and/or strawberries) and 1% fat milk. Intervention meals were prepared by a trained project manager and children were provided with a bar and a shake throughout the study period on the days they attended the afterschool program, typically 5 days per week (students received these meals instead of the standard meal). Students were provided with these meals for three months, with the length determined based on the previous literature of supplement trials in children and to minimize loss to follow-up [15]. Overall, these intervention meals provided approximately 75% of the FDA Daily Values for all essential vitamins and minerals, while the standard meals were not fortified. The total calories in the standard and intervention meals were similar (average 560 kcal).

### 2.3. Cognitive Measures

The cognitive measures used in the study included well-established assessments for children in the study age range. An estimate of IQ was determined using the Shipley-2 Composite Standard Score (based on both the Shipley Vocabulary and Abstraction scores), which measures crystallized and fluid cognitive abilities via questions requiring participants to correctly define words (Vocabulary) and solve problems using logic (Abstraction) [17]. The Stroop Color Word Test (Golden version) was used to evaluate executive functioning, selective attention, and processing speed [18]. The Stroop includes three conditions where participants are asked to (1) read color words printed in black ink (i.e., red, green, blue); (2) name colored blocks; and (3) name the color of words printed in an incongruous ink color (e.g., the word “red” is printed in green ink). An interference T-score is then derived based on performance across the three conditions with higher scores indicative of better performance. The Trail Making Test was used to measure psychomotor speed (Part A) and cognitive set shifting (Part B). During Part A, participants are required to draw lines connecting numbers while during Part B they must alternate between connecting numbers and letters (1, A, 2, B, etc.) [19]. Faster completion times with fewer errors reflect better performance on both Part A and B of the Trail Making Task. Alternative versions of the Trail Making Task were used at baseline and post-intervention. Lastly, the Conners Continuous Performance Test 3rd Edition (CPT) was administered to examine attention, impulsivity, and vigilance [20]. The CPT is a computerized task in which participants are required to press a button when any letter except “X” appears on the screen; several dependent variables are calculated regarding performance on the task. Detectability serves as a measure of how well participants discriminate non-targets (“X”) from targets (all other letters); higher T-score values indicate worse performance. Omissions errors are missed targets, whereas commission errors are incorrect responses to non-targets; higher rates of omissions and commissions are denoted by higher T-scores. Higher omission rates generally indicate inattentiveness, and while increased commission errors may also indicate inattentiveness, they are also reflective of impulsivity if observed in the context of faster response times. Perseverations are responses that that occur too quickly to be considered a valid response; higher scores are potentially associated with impulsivity. In addition, hit reaction time (HRT) T-scores reflect the mean response speed for correct responses, with a very fast HRT (lower T-score) potentially indicating impulsivity, and a very slow HRT (higher T-score) may be reflective of inattentiveness. Finally, HRT block change is a measure of the change in HRT across the six blocks of the administration; higher scores indicate potential losses of sustained attention. All cognitive tests were administered by trained research assistants, blind to students’ intervention status. Students were not blinded to the intervention as there was no comparable control food.

### 2.4. Procedures

Baseline testing began in January 2018. The Stroop, Trail Making Test, and CPT were administered during the afterschool program between approximately 3 and 5 p.m. Following baseline completion of the cognitive tests, students randomized to the intervention arm began receiving the intervention meals daily whereas the control arm continued to receive standard meals provided by the afterschool program. Follow-up cognitive testing occurred in May 2018 after approximately three months of either intervention or control meals consumption. The time and day of the week of administration of the tests pre- and post-intervention in the afternoon were matched as closely as possible. Compliance with consumption of the meals offered was also measured daily by a research assistant using validated visual estimation techniques (quarter-waste method) to determine the approximate percentage of the meals consumed (average consumption per student) [21]. Briefly, a trained research assistant compared each remaining food item with a corresponding control (i.e., unconsumed) sample and estimated if none, ¼, ½, ¾, or all of the item was consumed. This study was approved by Merrimack College Institutional Review Board (protocol# IRB-FY17-18-159).

### 2.5. Statistical Analysis

In order to assess between-group differences for demographic variables, analyses of variance (ANOVAs) were performed on scale data (i.e., age and IQ), and chi-squared independence tests were performed on nominal data (i.e., sex and ethnicity). These analyses indicated that the intervention group was significantly older than the control group. Therefore, for the Stroop and CPT, age was controlled for using conventional scoring techniques in which raw scores were transformed into age-corrected T-scores. To control for age on the Trail Making Test, raw scores were analyzed using analyses of covariance (ANCOVA) with age entered as a covariate. ANOVAs also indicated that children in the control condition ate significantly more of the standard meals than the children in the intervention condition consumed of the fortified meals; this difference was controlled for in secondary ANCOVAs which included meal consumption as a covariate. Otherwise, the groups were well-matched on all other demographic variables. Accordingly, in order to examine differences in cognitive scores, 2 × 2 mixed model ANOVAs (Stroop and CPT) or ANCOVAs (Trail Making Test) were performed. These analyses assessed the main effects of group (control vs. intervention) and time (baseline vs. post intervention) and examined interaction effects (group*time) which reflect differences between the groups over time. Independent samples *t* tests were also performed post hoc to assess whether the groups differed from one another at baseline or at follow-up (examined at both *p* ≤ 0.05 and *p* ≤ 0.10).

## 3. Results

For the final sample of 35 students who completed the study, the average age of the participants in the control group was 9.6 years (SD = 0.8) compared with 10.4 years in the intervention group (SD = 0.8; *F*(1,33) = 7.15, *p* = 0.01 (Table 1)). On average, 43.8% were female in the control group and 73.7% were female in the intervention group, although this difference was not statistically significant (*X*^2^(1, *N* = 35) = 3.24, *p* = 0.07). Nearly all participants were Hispanic, with 93.8% reporting that as their race/ethnicity in the control group and 100.0% in the intervention group (*X*^2^(1, *N* = 35) = 1.22, *p* = 0.27). There were no significant differences in the baseline Shipley IQ scores between the control and intervention groups (87.9 vs. 88.1; *F*(1,20) < 0.01, *p* = 0.97). Children ate on average more of the standard control meals than the intervention meals (88.8% vs. 76.5%; *F*(1,33) = 8.54, *p* = 0.01); this difference was controlled for in secondary analyses.

When examining differences on the cognitive tests over time, several significant differences were detected (Table 2). For the interference T-score of the Stroop task, a significant main effect of time was observed, indicating improvement over time in both the control and intervention groups (*F*(1,28) = 13.79, *p* < 0.01). Similarly, on the Trail Making Task, a significant main effect of time indicated that both groups exhibited fewer errors on Trails B at follow-up compared to baseline (*F*(1,27) = 9.92, *p* < 0.01). On the CPT a significant main effect of time indicated increased omission errors at follow-up relative to baseline (*F*(1,23) = 7.00, *p* = 0.01) in both groups. However, a comparison of the group means at follow-up indicated a trend for the control group to make more CPT omission errors relative to the intervention group (*t*(28) = 1.46, *p* = 0.08) indicating that the main effect of time was primarily driven by poorer performance in the control group. No significant main effects of group or group*time interactions were observed.

In a secondary analysis, outliers, defined as values beyond 1.5× the interquartile range, were removed from analyses and meal consumption was included as a covariate using ANCOVAs. The outlier analyses identified participants who performed more poorly than their peers including 2 participants as outliers on the Stroop task (Control *n* = 1, Intervention *n* = 1), 5 participants on the Trail Making Task (Control *n* = 3, Intervention *n* = 2) and 4 participants on the CPT (Control *n* = 2, Intervention *n* = 2). Results from ANOVA assessments with performance outliers removed indicated that the main effects of time for Stroop, Trails B errors, and CPT omissions remained significant; however, when meal consumption was included as a covariate, only the main effect of time for Trails B errors remained significant (Table 3).

## 4. Discussion

To our knowledge, this is the first randomized controlled feeding trial to examine the impact of a fortified food intervention containing all essential vitamins and minerals, as well as macronutrients, and fruit on cognitive functioning among low-income children in the United States. This pilot study found that both the intervention and control group improved on some of the cognitive tests (i.e., Stroop and Trail Making Test) over the three month intervention, but there were no meaningful differences in scores between the two groups over time Additionally, when the amount of the meal consumed was included as a covariate in the analyses, almost all results became non-significant, suggesting that food consumption has a significant impact on the current findings.

The results of this study are similar to other vitamin and mineral supplement trials in children. A randomized control trial conducted among 86 school children aged 11–13 in the UK that administered vitamin and mineral supplements (with varying RDAs for Iron, Calcium, Zinc, Magnesium, Phosphorous, Vitamin A, Thiamin, Riboflavin, Niacin, Vitamin B6, Vitamin B12, Folate, Vitamin C, Vitamin D, Vitamin E) for seven months did not observe significant differences in cognitive functioning scores (verbal and non-verbal reasoning) over time similar to the current study [22]. A second randomized controlled trial among 227 children aged 7–12 years similarly found no significant differences in verbal and non-verbal intelligence scores over time after 28 days of vitamin and mineral supplementation (which included varying amounts of Calcium, Iron, Zinc, Chromium, Copper, Iodine, Magnesium, Manganese, Selenium, Vitamin A, Thiamin, Riboflavin, Vitamin B6, Vitamin B 12, Biotin, Pantothenic acid, Vitamin C, Vitamin D, Vitamin E, and Vitamin K). [23]. This contrasts with the results of several other vitamin and mineral supplement trials in children which reported significant positive associations with cognitive performance [15]. These studies tended to be longer in duration (5 months to 1 year), although there were studies as short as 7 weeks that found positive associations with cognitive functioning while others that were over 30 weeks in duration found no association [15]. Heterogenous results in these studies may, therefore, be due to methodological differences, including variation in cognitive assessment measures, micronutrients provided, and sample size, as well as subtle but important differences in the populations being studied such as age, race, and/or socioeconomic status of the participants.

This study had several limitations. First, on average, participating students had below average IQs, and it is, therefore, possible that this impacted performance on other cognitive measures. However, these lower scores are also possibly the result of using the Shipley test, which only provides an estimate of IQ, unlike other, more comprehensive tests, and/or may have been influenced by potential test-based cultural biases towards minorities or low-income individuals [24]; therefore this measure may have underestimated their IQs. Importantly, the intervention and control groups had similar IQ scores at baseline, indicating similar levels of intelligence between the two groups prior to the study intervention. Furthermore, despite randomization, the intervention group was slightly older on average compared to the control group; however, this age difference was controlled for in the statistical analyses through the use of age-adjusted T-scores or utilization of ANCOVAs. Further, this study was conducted among low-income, predominately Hispanic children aged 8–12 years, and, therefore, the results may not be generalizable to other children, including those who are younger or adolescents. The study may have also been limited by its relatively small sample size, which may have been insufficient to detect subtle changes in cognition. Additionally, the students participating in the study could not be blinded to their intervention status, which may have impacted their performance. In order to limit potential bias during testing, the research assistants administering the cognitive tests were blind to the students’ intervention status. Students also consumed significantly less of the intervention meals compared with the control meals, which highlights the need for future studies to also consider the palatability of the foods provided to children. As benefits were seen with increased overall consumption, this may highlight the need to ensure potentially food-insecure children are provided with foods that are both nutritious and taste good. Other residual confounding variables may have impacted study results. As neither testing nutrient deficiencies nor validly assessing the participants’ overall diets was feasible due to both their age and potential issues among a food insecure population, it is also possible that the participants had sufficient nutrient intakes at baseline, although these children were from low-income and/or food-insecure households. Lastly, because only one meal was provided daily, it is possible that the participants’ food consumption outside of the afterschool meal program impacted the study results. Strengths of the study included the ability to randomize students to different meals, high compliance with the assigned diets, and reducing the potential for practice effects associated with repeated administration of cognitive tasks by utilizing alternative versions of the Trail Making Task as well as employing a long interval between testing sessions.

## 5. Conclusions

Overall, results from this pilot study suggest that there may be limited additional benefits to short-term consumption of micronutrient fortified meals provided in an afterschool care setting to a low-income population; however, any benefits may be directly related to the amount of food consumed. Future research should examine whole food approaches, as well a larger feeding trials in this population for longer durations with additional assessments of nutritional deficiencies.

## Figures and Tables

**Table 1:** Characteristics of Children Participating in the Feeding Trial ^1^.

	Control (*n* = 16) Mean (SD)	Intervention (*n* = 19) Mean (SD)	Significance *p*
	Baseline		
Age	9.6 (0.8)	10.4 (0.8)	0.01
Sex: Female (%)	43.8	73.7	0.07
Race/Ethnicity: Hispanic (%)	93.8	100.0	0.27
Shipley Estimated IQ ^2^	87.9 (10.7)	88.1 (10.8)	0.97
	During Intervention Period		
Average Study Meal Consumption (%)	88.8 (7.9)	76.5 (15.2)	**<0.01**

Significant effects at *p* ≤ 0.05 are indicated in bold. ^1^ Children were randomized to control (i.e., standard) meals or intervention meals (i.e., bars and shakes fortified with all essential vitamins and minerals, omega-3 and omega-6 fatty acids, protein, and fruit). ^2^ The Shipley Composite Standard Score for IQ is based on both the Shipley Abstraction and Vocabulary scores.

**Table 2:** Cognitive Scores among Students Participating in a feeding trial ^1^ at baseline and post-implementation: Analyses of Variance (ANOVA) Results.

	Control Group		Intervention Group		ANOVA					
	Baseline Mean (SD)	Post Mean (SD)	Baseline Mean (SD)	Post Mean (SD)	Main Effect: Group		Main Effect: Time		Interaction: Group*Time	
					*F*	*p*	*F*	*p*	*F*	*p*
**Stroop Color Word Task T-Scores**										
Interference	42.6 (9.5)	49.6 (9.1)	45.5 (7.0)	49.1 (6.1)	0.20	0.66	**13.79**	**<0.01**	1.46	0.24
**Trail Making Task** ^2^										
Trails A Time (s)	51.9 (23.3)	53.8 * (34.1)	43.8 (16.2)	31.2 * (20.4)	3.41	0.07	0.04	0.85	1.34	0.26
Trails A Errors	2.3 (2.7)	1.7 ^ (1.3)	2.1 (1.6)	1.1 ^ (1.2)	0.07	0.80	0.06	0.82	0.21	0.65
Trails B Time (s)	172.8 * (84.0)	128.9 * (97.3)	126.2 * (63.5)	85.7 * (63.5)	2.26	0.14	2.33	0.14	0.20	0.66
Trails B Errors	3.9 (3.4)	1.9 (1.7)	2.9 (2.7)	1.9 (1.8)	0.05	0.83	**9.92**	**<0.01**	0.70	0.41
**Conner’s Continuous Performance Task T-Scores**										
Detectability	59.9 (8.8)	64.3 ^ (11.8)	56.3 (7.8)	55.9 ^ (9.6)	2.95	0.10	1.32	0.26	1.84	0.19
Omissions	58.5 (12.8)	70.4 ^ (15.2)	57.6 (12.8)	60.7 ^ (13.2)	1.27	0.27	**7.00**	**0.01**	2.43	0.13
Commissions	57.6 ^ (9.4)	54.4 (10.0)	52.6 ^ (7.5)	50.9 (8.3)	1.58	0.22	*3.08*	0.09	0.28	0.60
Perseverations	62.9 * (15.7)	62.1 (16.7)	49.6 * (6.8)	59.5 (16.2)	2.71	0.11	1.77	0.20	2.37	0.14
Hit Reaction Time (HRT)	53.1 ^ (8.8)	59.9 (14.3)	58.3 ^ (8.8)	59.5 (14.5)	0.41	0.53	1.80	0.19	0.84	0.37
HRT Block Change	49.5 (17.6)	58.0 (12.4)	57.1 (9.2)	58.0 (13.2)	1.01	0.33	1.34	0.26	0.87	0.36
HRT Inter-Stimulus Change	54.3 (8.5)	58.9 (16.4)	53.5 (7.0)	55.5 (7.6)	0.56	0.46	0.95	0.34	0.13	0.72

Significant effects at *p* ≤ 0.05 are indicated in bold. ^1^ Children were randomized to control (i.e., standard) meals or intervention meals (i.e., bars and shakes fortified with all essential vitamins and minerals, omega-3 and omega-6 fatty acids, protein, and fruit). ^2^ Results are not T-scores, and, therefore, age is controlled for using analyses of covariance (ANCOVAs). * Indicates significant between-group differences at the baseline or post assessment at *p* ≤ 0.05. ^ Indicates significant between-group differences at the baseline or post assessment at *p* ≤ 0.1.

**Table 3:** Cognitive Scores among Students Participating in a feeding trial ^1^ at baseline and post-implementation: Analyses of Co-Variance (ANCOVA) Results Controlling for Amount of Meals Consumed and with Performance Outliers Removed.

	Control Group		Intervention Group		ANCOVA (Controlling for Meal Consumption)					
	Baseline Mean (SD)	Post Mean (SD)	Baseline Mean (SD)	Post Mean (SD)	Main Effect: Group		Main Effect: Time		Interaction: Group*Time	
					*F*	*p*	*F*	*p*	*F*	*p*
**Stroop Color Word Task T-Scores**										
Interference	40.9 * (6.9)	48.2 (7.6)	46.4 * (6.2)	50.0 (5.0)	2.45	0.13	0.11	0.75	0.38	0.55
**Trail Making Task ^2^**										
Trails A Time (s)	46.3 (16.3)	47.0 (32.3)	46.4 (15.0)	31.8 (21.3)	0.77	0.39	<0.01	0.96	2.79	0.11
Trails A Errors	1.5 (1.6)	1.6 (1.4)	2.1 (1.6)	1.0 (1.2)	0.26	0.61	0.25	0.62	2.65	0.12
Trails B Time (s)	156.9 (77.6)	107.1 (69.3)	132.9 (63.8)	88.1 (66.8)	0.04	0.84	1.04	0.32	0.26	0.62
Trails B Errors	3.3 (3.2)	1.4 (1.6)	2.9 (2.8)	2.1 (1.9)	1.79	0.20	**4.80**	**0.04**	0.63	0.44
**Conner’s Continuous Performance Task T-Scores**										
Detectability	61.9 * (6.5)	64.5 (9.1)	56.0 * (6.0)	57.7 (9.1)	2.92	0.11	1.01	0.33	0.44	0.52
Omissions	61.3 * (11.7)	72.2 ^ (13.3)	55.1 * (7.5)	59.3 ^ (8.6)	*3.23*	0.09	1.46	0.24	1.96	0.18
Commissions	59.2 * (7.4)	55.6 (5.9)	53.3 * (6.4)	53.0 (6.1)	2.11	0.16	0.39	0.54	0.28	0.60
Perseverations	64.2 * (16.2)	64.6 (16.7)	49.6 * (7.6)	61.9 (16.9)	0.57	0.46	0.09	0.78	1.50	0.24
Hit Reaction Time (HRT)	54.0 (9.2)	58.3 (14.9)	56.3 (7.0)	58.2 (13.9)	0.13	0.73	0.17	0.70	0.20	0.66
HRT Block Change	50.3 (19.2)	55.5 (8.6)	58.4 (9.7)	60.2 (13.1)	1.35	0.26	0.63	0.44	0.44	0.52
HRT Inter-Stimulus Change	55.7 (8.2)	56.3 (14.9)	52.3 (7.2)	55.1 (7.9)	0.06	0.81	0.19	0.67	0.01	0.95

Significant effects at *p* ≤ 0.05 are indicated in bold. ^1^ Children were randomized to control (i.e., standard) meals or intervention meals (i.e., bars and shakes fortified with all essential vitamins and minerals, omega-3 and omega-6 fatty acids, protein, and fruit). ^2^ Results are not T-scores, and, therefore, age and meal consumption are controlled for in the ANCOVAs. * Indicates significant between-group differences at the baseline or post assessment at *p* ≤ 0.05. ^ Indicates significant between-group differences at the baseline or post assessment at *p* ≤ 0.10.

## Supplementary Materials

The following are available online at https://www.mdpi.com/2072-6643/12/11/3351/s1, Table S1: Average nutrient values for intervention and standard meals provided to students participating in a feeding trial.
