# Supplementary material for: The Impact of Micronutrient Fortified Foods on Cognitive Functioning among Low-Income Children: A Pilot and Feasibility Study
Source: Nutrients. 2020 Oct 30;12(11):3351. doi: 10.3390/nu12113351 (PMC7693551; doi:10.3390/nu12113351)
Supplement: Supplementary file 1 [file nutrients-12-03351-s001.pdf]

Supplemental Table 1. Average Nutrient Values for Intervention and Standard Meals Provided to Students Participating in a Feeding Trial

	Intervention Meals	Standard Meals
	Average	
Calories (kcal)	570	550
Total Fat (g)	14	19
Saturated Fat (g)	3.5	7
Sodium (mg)	245	615
Carbohydrates	77	76
Dietary Fiber (g)	14	1
Protein (g)	34	19
	Average based on % Daily Values	
Biotin	75	13
Calcium	85	9
Choline	75	15
Chromium	75	6
Copper	75	17
Folate	90	18
Iodine	75	1
Iron	75	23
Magnesium	85	9
Manganese	100	0
Molybdenum	75	18
Niacin	75	31
Pantothenic Acid	75	12
Phosphorous	95	19
Riboflavin	80	17
Selenium	75	33
Thiamin	75	22
Vitamin A	75	6
Vitamin B6	75	16
Vitamin B12	80	62
Vitamin C	100	6
Vitamin D	80	2
Vitamin E	75	4
Vitamin K	100	18
Zinc	75	16
